# Gut microbial evidence chain in high-salt diet exacerbates intestinal aging process

**DOI:** 10.3389/fnut.2022.1046833

**Published:** 2022-10-28

**Authors:** Tian-hao Liu, Lin Zhao, Chen-yang Zhang, Xiao-ya Li, Tie-long Wu, Yuan-yuan Dai, Ying-yue Sheng, Yi-lin Ren, Yu-zheng Xue

**Affiliations:** ^1^Department of Gastroenterology, Affiliated Hospital of Jiangnan University, Wuxi, China; ^2^Wuxi School of Medicine, Jiangnan University, Wuxi, China; ^3^College of Chinese Medicine, Hunan University of Chinese Medicine, Changsha, China

**Keywords:** high-salt diet, intestinal aging, gut microbiota, machine learning, signal transduction

## Abstract

Although excessive salt consumption appears to hasten intestinal aging and increases susceptibility to cardiovascular disease, the molecular mechanism is unknown. In this study, mutual validation of high salt (HS) and aging fecal microbiota transplantation (FMT) in C56BL/6 mice was used to clarify the molecular mechanism by which excessive salt consumption causes intestinal aging. Firstly, we observed HS causes vascular endothelial damage and can accelerate intestinal aging associated with decreased colon and serum expression of superoxide dismutase (SOD), glutathione peroxidase (GSH-Px), and increased malondialdehyde (MDA); after transplantation with HS fecal microbiota in mice, vascular endothelial damage and intestinal aging can also occur. Secondly, we also found intestinal aging and vascular endothelial damage in older mice aged 14 months; and after transplantation of the older mice fecal microbiota, the same effect was observed in mice aged 6–8 weeks. Meanwhile, HS and aging significantly changed gut microbial diversity and composition, which was transferable by FMT. Eventually, based on the core genera both in HS and the aging gut microbiota network, a machine learning model was constructed which could predict HS susceptibility to intestinal aging. Further investigation revealed that the process of HS-related intestinal aging was highly linked to the signal transduction mediated by various bacteria. In conclusion, the present study provides an experimental basis of potential microbial evidence in the process of HS related intestinal aging. Even, avoiding excessive salt consumption and actively intervening in gut microbiota alteration may assist to delay the aging state that drives HS-related intestinal aging in clinical practice.

## Introduction

Aging is an unavoidable process in human life activities that involves the gradual decline in the function of various organ systems, which is reflected at the cellular, tissue, organ and systemic levels and further leads to the onset, development and death of many diseases (1, 2). Even, aging is a risk factor for chronic diseases, among others, senescence mechanisms have become a target of huge research on the topic of the aging process (1). Intestinal aging is an important element of the process, and it is also said that aging starts in the gut. As the aging process is accompanied by the accumulation of damage to the body, it leads to reduced function and vulnerability to disease (3, 4). The physiological and immune functions of the intestine gradually decline with increasing age, and the structure of the diet and gut microbiota also change accordingly (5). To our knowledge, aging is accompanied by degenerative changes in the intestinal tract, including histopathological changes in the intestinal tract, weakened intestinal contraction and peristalsis, reduced secretion, decreased levels of various digestive enzymes, resulting in reduced intestinal digestion and absorption, reduced gut microbiota, increased intestinal permeability, causing intestinal dysfunction and chronic inflammation of the intestinal tract (6).

Only a few of the various processes that make up the complex process of aging and act as important entry points for age-related diseases include immunosenescence and inflammaging. Additionally, diet, prebiotics, probiotics, and synbiotics may extend longevity through gut microbiota manipulation (7). The microbiota-targeted dietary and probiotic interventions have been shown to favorably affect the host health and aging by an enhancement of antioxidant activity, improving immune homeostasis, suppression of chronic inflammation, regulation of fat deposition and metabolism and prevention of insulin resistance. The function of gut microbiota in aging processes is discussed in recent research findings, with a focus on the therapeutic potential of microbiome-targeted therapies in anti-aging therapy (8).

High salt (HS) is a risk factor for a variety of diseases, and HS may play a role in the development of gastrointestinal disorders *via* intestinal microenvironmental remodeling, adding to a better understanding of HS's complicated pathogenic function in gastrointestinal diseases (9). Thus, HS is a major cause of many chronic and age-related deficiencies such as cardiac hypertrophy, exercise disorders, and death (10). Growing data suggests that the gut microbiota is at the root of many age-related changes, such as immune system dysregulation and disease vulnerability. Throughout its life cycle, the gut microbiota has undergone significant changes, and aging-related processes may have an impact on the gut microbiota and the metabolic changes that go along with it (11). The connection between HS, the gut microbiota, and intestinal aging hasn't been well explored, though. To accomplish this, we employed older animals for further verification after performing 16S rRNA gene sequencing on bacterial DNA taken from HS-induced mice and preliminary verification using FMT of HS-induced mice. To prove that HS promotes intestinal aging through the gut microbiota, fecal microbiota were implanted in older mice. We looked at relationships between these microbial fingerprints and biological age related microbiota genera, which are excellent predictors of mortality, morbidity, and other age-related events.

## Methods

### Design and grouping

A total of 24 C56BL/6 mice (6–8 weeks old, male, provided by Changzhou cavens experimental animal Co., Ltd.) were randomly divided into normal control group (CON), high salt group (HS) and fecal microbiota transplantation (FMT) of HS group mice to normal control mice group (FMT-HS). Meanwhile, sixteen 14-month-old C56BL/6 mice (male, provided by Changzhou cavens experimental animal Co., Ltd.) were randomly designed as older control group (CON-O) and fecal microbiota of older mice transplanted to normal control mice group (FMT-O), with 8 mice in each group for experimental validation. The ethics committee of Jiangnan University approved the animal experiment which was carried out in the university's animal facility (NO. 20211015c0650220).

### FMT preparation

Fresh feces were collected from HS group mice and stored into a 50 ml centrifuge tube every day. Then the collected feces were dissolved using normal saline in the ratio of 1:10 according to our previous studies (12). Thus, the fecal samples were centrifuged (3,000 rpm for 5 min) after sufficient mixing. The fecal bacterial suspension was transferred and gained into a sterile centrifuge tube and then were administered by gavage to mice within 2 h (12).

### Intervention

In this study, an 8% HS (NaCl) diet for 4 weeks was used in the HS group, while an 0.4% salt (NaCl) diet was used in other groups. Nantong Troffer Feed Technology Co., Ltd. (Nantong, China) provided the HS feed [production license: (2014) 06092], which was then sterilized by Nantong Michael Irradiation Co., Ltd. (Nantong, China). The mice in FMT-HS and FMT-O groups were respectively gavaged using FMT of the mice in HS group mice or older control group (100 μl/d per mouse), while the mice in other groups were administered by gavage with 100 μl normal saline.

### Measurement of intestinal aging-related factors in colon and serum by enzyme-linked immunosorbent assay

To reduce suffering, isoflurane anesthesia was administered to all mice after 4 weeks. After that, blood was extracted from the eyeball and centrifuged after 2–4 h for 5 min at 3,000 rpm. The supernatant was then sub packed for storage into 1.5-ml sterilized EP tubes. The colon tissue (1–2 cm) was gathered and stored in the EP tubes. Lastly, the levels of malondialdehyde (MDA), catalase (CAT), glutathione peroxidase (GSH-Px), and superoxide dismutase (SOD) in the colon and serum were assessed in accordance with the kit's instructions, which were bought from MEIMIAN (Yancheng, China).

### Detection of vascular endothelial function-related factors in serum by ELISA

Then, using kits given by MEIMIAN (Yancheng, China) and following the operating instructions, the levels of nitric oxide (NO), endothelin-1 (ET-1), angiotensin II (AngII), vascular endothelial growth factor (VEGF), and vitamin k2 (VK2) were determined.

### 16S rRNA gene sequencing, gut microbial analysis of intestinal contents

The intestinal contents of all mice were stored, and the intestinal contents of 6 mice in each group were randomly selected for subsequent analysis. The intestinal contents were used to extract microbial DNA through E.Z.N.A.^®^ soil DNA Kit (Omega Bio-Tek, Norcross, GA, U.S.). The final DNA concentration and purification was assessed using NanoDrop 2000 UV-vis spectrophotometer (Thermo Scientific, Wilmington, USA), and DNA quality was estimated using 1% agarose gel electrophoresis. The V3-V4 hypervariable portions of the bacterium 16S rRNA gene were amplified by a thermocycler PCR system (GeneAmp 9700, ABI, USA) with primers 338F (5′-ACTCCTACGGGAGGCAGCAG-3′) and 806R (5′-GGACTACHVGGGTWTCTAAT-3′). The PCR products were extracted from a 2% agarose gel, purified with a AxyPrep DNA Gel Extraction Kit (Axygen Biosciences, Union City, CA, USA), and quantified with QuantiFluorTM-ST (Promega, USA) according to the manufacturer's instructions.

The purified amplicons were sequenced on an Illumina MiSeq platform (Illumina, San Diego, USA) at an equimolar ratio (Shanghai, China). The operational taxonomic units (OTUs) were clustered using UPARSE (http://drive5.com/uparse/) with a unique “greedy” technique that performs chimera filtering and OTU clustering at the same time. Finally, the RDP Classifier algorithm was used to compare the taxonomy of each 16S rRNA gene sequence to the 16S rRNA database [Silva (SSU123)].

### Prediction model for intestinal aging based on microbiota genera with relative abundance difference

To develop a system for diagnosing intestinal aging, we explored a machine learning technique based on the significantly different microbiota. Machine learning is the process through which computer systems gradually increase their capacity to execute specified tasks by using computer algorithms and statistical models (12). In order to generate predictions or judgments without explicitly programming to carry out tasks, machine learning creates a mathematical model using sample data, known as “training data.” In this work, a model of intestinal aging was created using the LDA linear judgment analysis approach. CON-O and CON group made up the necessary training set for the modeling process. After that, a different set of samples was utilized as a test set to assess the model's capacity to detect intestinal aging brought on by excessive salt intake. The mass package of the R language were used to analyze this section.

### Statistical analysis

GraphPad Prism 8.3 was used to analyze all data, which were all expressed as mean standard error (SEM). The Kruskal-Wallis test or one-way ANOVA were used for all analyses. *P* < 0.05 denotes a meaningful difference. The Majorbio I-Sanger Cloud Platform was utilized to examine the 16S rRNA gene sequencing data (www.i-sanger.com).

## Results

### HS induced intestinal aging process in mice related to gut microbiota

**Figure 1:**
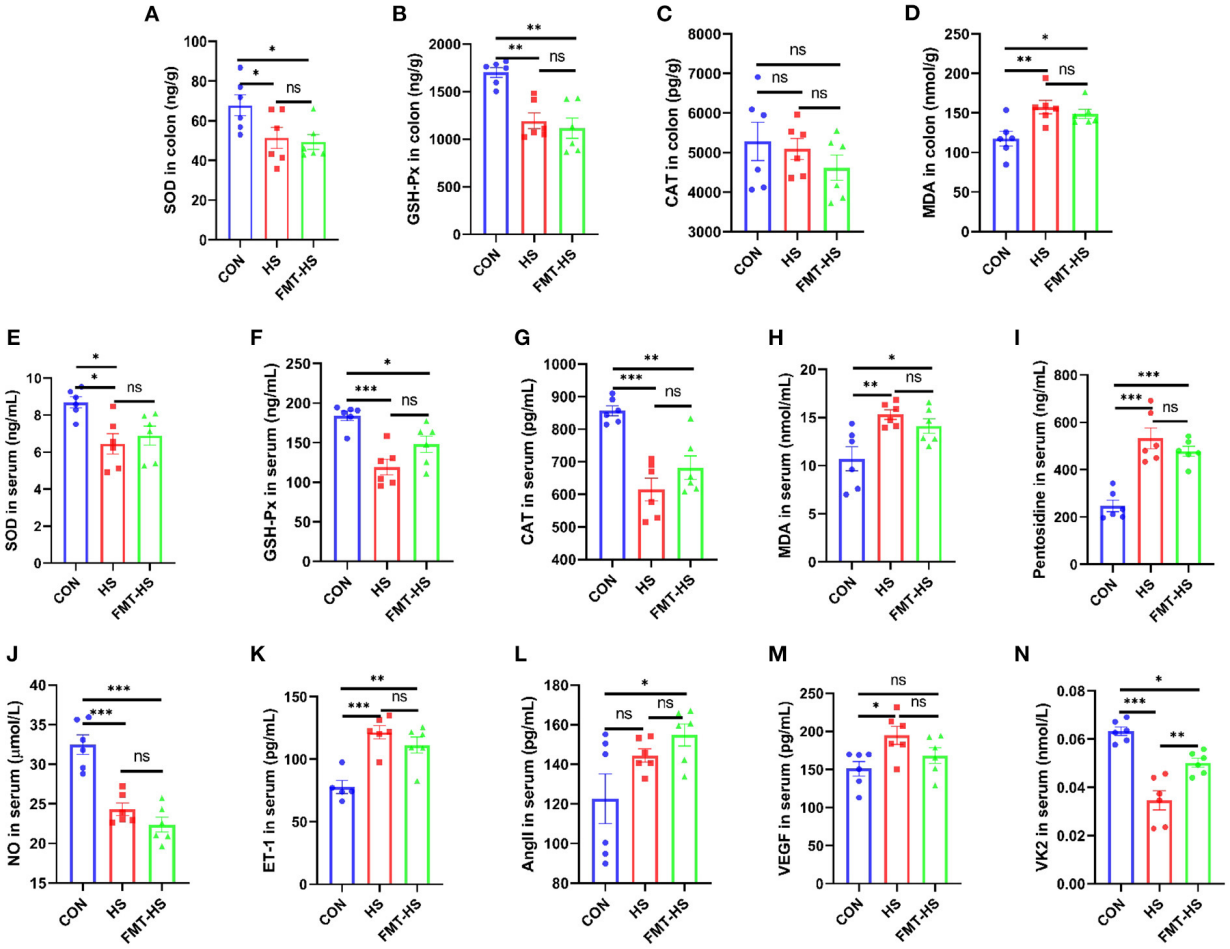
High salt (HS) induced intestinal aging-related factors imbalance in mice, which was transferable by fecal microbiota transplantation. The changes of aging-related factors in colon and serum **(A–I)**. The changes of vascular endothelial function-related factors in serum **(J–N)**. SOD, superoxide dismutase; GSH-Px, glutathione peroxidase; CAT, catalase; MDA, malondialdehyde; NO, nitric oxide; ET-1, endothelin-1; AngII, angiotensinII; VEGF, vascular endothelial growth factor; VK2 vitmain k2, respectively. CON, natural diet; HS, 8% salt diet; FMT-HS, gut microbiota of HS group mice transplanted to CON group mice. All data are expressed as mean ± standard error (SEM). One-way ANOVA with Tukey *post hoc* test was conducted. *p < 0.05, **p < 0.01, ***p < 0.001; ns, no significance.

We first measured intestinal aging-related factors (SOD, GSH-Px, CAT, MDA, pentosidine) in colon and serum in CON, HS, FMT-HS groups (Figures 1A–I). As expected, significantly decreased levels of SOD and GSH-Px, significantly increased level of MDA in colon were found in HS group after HS induced for 4 weeks, which were also found in FMT-HS group mice (Figures 1A,B,D). Nevertheless, no significant difference were found in the level of CAT in colon (Figure 1C). Meanwhile, significantly decreased levels of SOD, GSH-Px and CAT, significantly increased levels of MDA and pentosidine in serum were found in HS group after HS induced for 4 weeks, which were also found in FMT-HS group mice (Figures 1E–I). Thus, we measured the changes of vascular endothelial function-related factors (NO, ET-1, AngII, VEGF, VK2) in serum in CON, HS, FMT-HS groups (Figures 1I,J). Remarkably, significantly decreased levels of NO and VK2, significantly increased levels of ET-1, VEGF in serum were found in HS group after HS induced for 4 weeks, the significant changes in the levels of NO, VK2, ET-1, AngII, were found in FMT-HS group mice (Figures 1J–N). Nevertheless, increased trends were found in the level of AngII in serum in HS group and the level of VEGF in FMT-HS group (Figures 1L,M). These results suggest that HS induced intestinal aging process in mice related to gut microbiota.

### *HS* significantly changed gut microbial diversity, which was transferable by FMT

**Figure 2:**
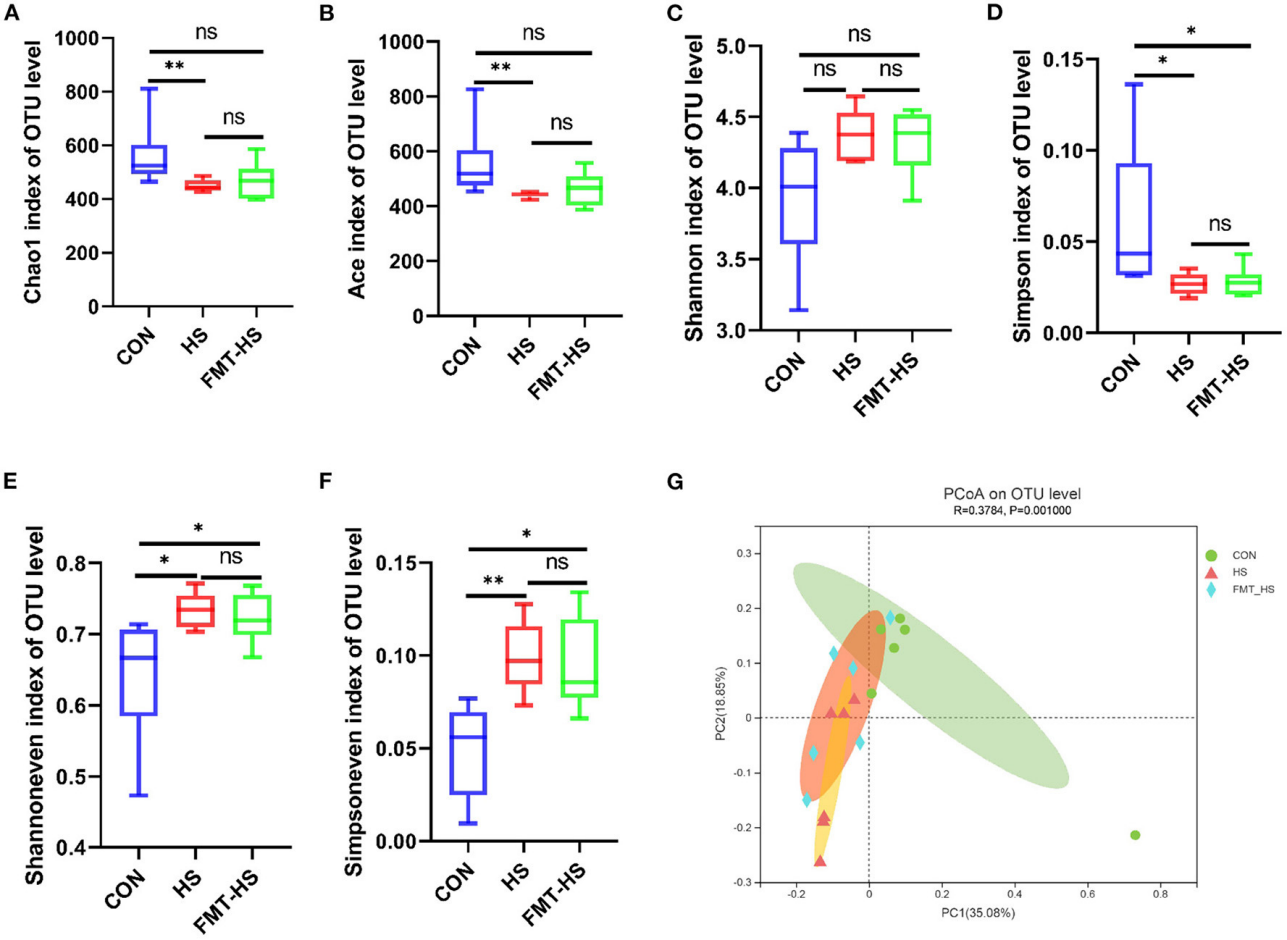
High salt (HS) dramatically altered gut microbial diversity, and fecal microbiota transplantation was able to transmit these changes. The changes of α-diversity **(A–F)**. PCoA plot in β-diversity **(G)**. The closer two sample points are, the more similar the composition of the two sample species. Different colored points and shapes indicate samples from different groups. All data are expressed as mean ± standard error (SEM). One-way ANOVA with Tukey *post hoc* test was conducted. *p < 0.05, **p < 0.01, **p < 0.001; ns, no significance.

To test how HS induced intestinal aging process in mice related to gut microbiota, we measured the changes in gut microbial diversity. As for gut microbiota, the alpha diversity were examined by indices such as chao1, ace, shannon, simpson, shannoneven and simpsoneven. Richness of the gut microbial community is reflected by the indices chao1 and ace, diversity of the gut microbial community is reflected by the indices shannon and simpson, and evenness of the gut microbial community is shown by the indices simpsoneven and shannoneven. The indices of chao1, ace, simpson in HS goup were found significantly lower than that of the CON group; the indices of shannoneven and simpsoneve were found significantly higher than that of the CON group (Figures 2A–F). While the index of simpson in FMT-HS group was also found significantly lower than that of the CON group, the indices of shannoneven and simpsoneven in FMT-HS group were also significantly higher than that of the CON group (Figures 2A–F). Nevertheless, no significant difference was found in the index of shannon (Figure 2C). The changes of the six indices indicate HS significantly changed gut microbial alpha diversity.

Beta diversity analysis is to explore the similarity or difference of community composition among different groups of samples by comparing and analyzing the species diversity among different habitats or microbial communities. Based on the principal co-ordinates analysis (PCoA), PC1 accounted for 35.08% of the total variation and PC2 accounted for 18.85%, which revealed the microbial community of the CON group differed significantly from that of the HS and FMT-HS groups (*R*^2^ = 0.3784, *p* = 0.001; Figure 2G). All these findings indicate HS induced intestinal aging process in mice related to gut microbial diversity.

### HS significantly changed gut microbial composition, which was screened by FMT

**Figure 3:**
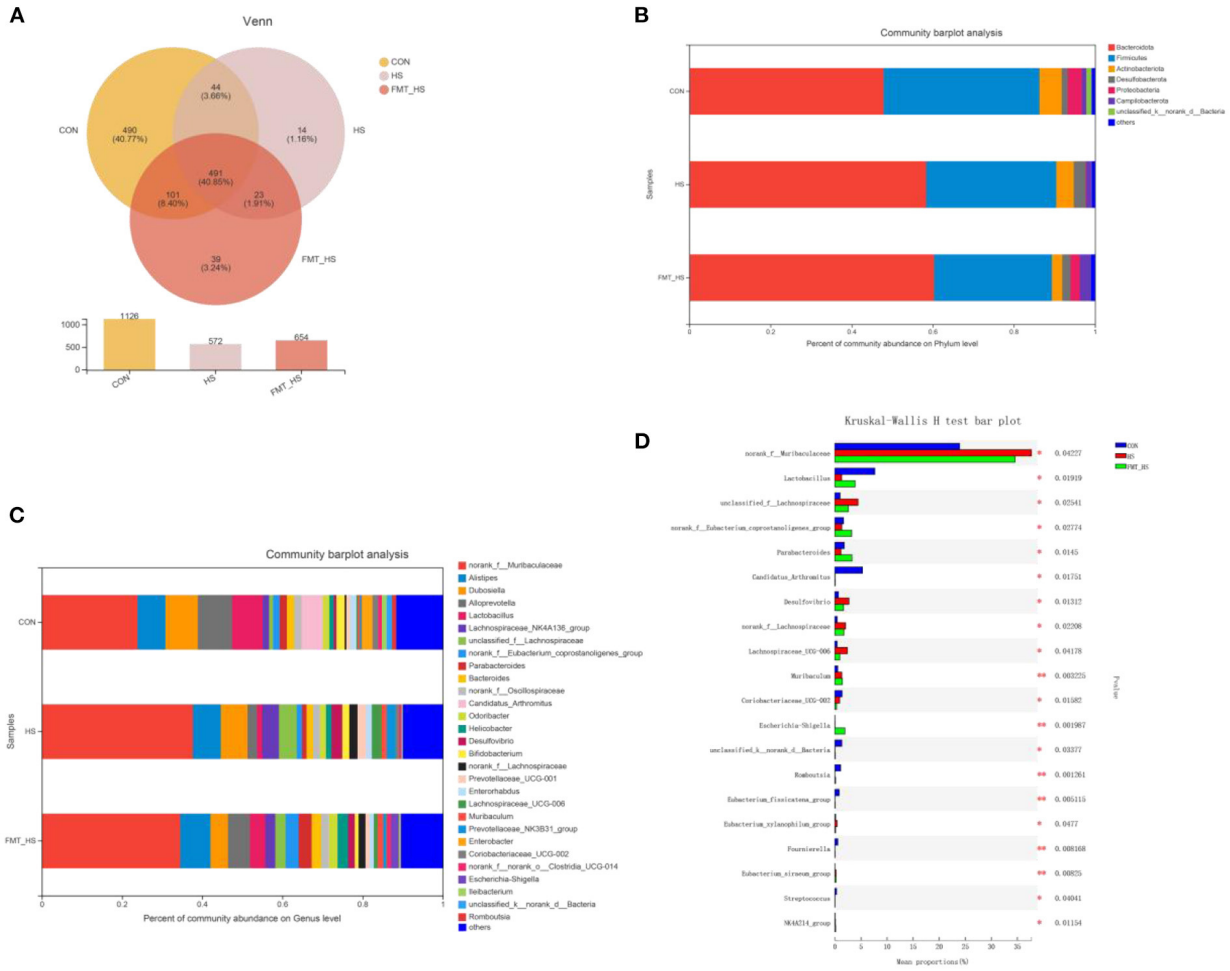
High salt (HS) significantly changed gut microbiota composition, which was screened by fecal microbiota transplantation (*n* = 6). The down-regulated OTU number is shown in here **(A)**. Phylum-level abundance as a percentage of total community abundance **(B)**. The top 30 in community abundance in terms of genera percent **(C)**. The significantly different genera **(D)**. Kruskal-Wallis test with Dunn *post hoc* test was used for statistical tests. *p < 0.05, **p < 0.01, **p < 0.001.

To explore how the microbial characteristics associate with the HS-related intestinal aging process, we calculated the differential relative abundance of characteristics among CON, HS and FMT-HS groups. Our study found that the CON group contained 1,126 OTUs, the HS group 572, and the FMT-HS group 654 (Figure 3A). The phyla-level and genera-level were then selected to perform further analysis (Figures 3B,C). As shown in Figure 3B, there were 38.43%, 32.04%, 29.04% Firmicutes in the CON, HS and FMT-HS group. There were 47.89%, 58.41%, 60.34% Bacteroidetes in the CON, HS and FMT-HS groups. In Figure 3C, the top 25 genera are displayed in the CON, HS, and FMT-HS groups. For example, there were 23.91%, 37.69%, 34.54% *norank_f__Muribaculaceae* in the CON, HS and FMT-HS groups; meanwhile, there were 8.07%, 6.67%, 4.34% *Dubosiella* in the CON, HS and FMT-HS groups. Our data indicate that HS can regulate the gut microbial community relative abundance, even increase Bacteroidetes and *norank_f__Muribaculaceae*, whereas decrease Firmicutes and *Dubosiella*. To investigate further how HS induced intestinal aging process in mice related to gut microbial composition, a total of 32 significantly different genera of the microbial community among the three groups were calculated (Supplementary Table 1), and the top 20 genera were shown as Figure 3D. Taken together, all these results reveal that HS induced intestinal aging process in mice related to gut microbial composition.

### Intestinal aging-related factors imbalance was also found in older mice, which was transferable by FMT

**Figure 4:**
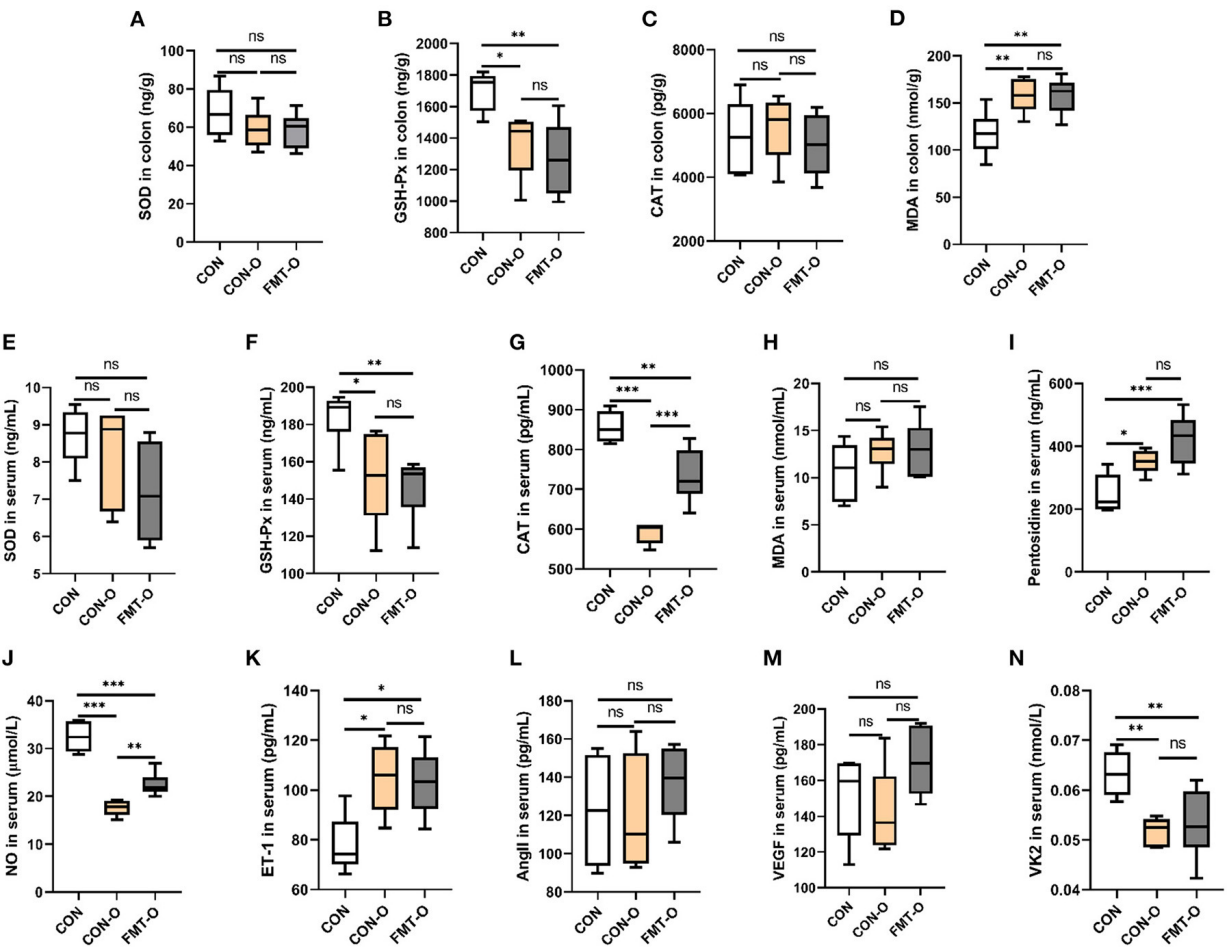
Intestinal aging-related factors imbalance was found in older mice, which was transferable by fecal microbiota transplantation. The changes of aging-related factors in colon and serum **(A–I)**. The changes of vascular endothelial function-related factors in serum **(J–N)**. SOD, superoxide dismutase; GSH-Px, glutathione peroxidase; CAT, catalase; NO, nitric oxide; ET-1, endothelin-1; AngII, angiotensinII; VEGF, vascular endothelial growth factor; VK2, vitmain k2, respectively. CON, natural diet; HS, 8% salt diet; FMT-HS, gut microbiota of HS group mice transplanted to CON group mice. All data are expressed as mean ± standard error (SEM). One-way ANOVA with Tukey *post hoc* test was conducted. *p < 0.05, **p < 0.01, ***p < 0.001; ns, no significance.

To verify whether HS induced intestinal aging process in mice related to gut microbiota, we explored the intestinal aging-related factors (SOD, GSH-Px, CAT, MDA, pentosidine) in colon and serum in CON, CON-O, FMT-O groups (Figures 4A–I). As seen in Figures 4A–I, the level of SOD in colon in CON group was lower than that in CON-O and FMT-O groups, however, there was no statistically significant difference. The level of GSH-Px in colon in CON group was significantly higher than that in CON-O and FMT-O groups. No statistically significant difference was found in the level of CAT in colon among the three groups. In addition, the levels of GSH-Px and CAT in serum in CON group were found significantly higher than that in CON-O and FMT-O groups (Figures 4F,G). The level of pentosidine in colon in CON group was found significantly lower than that in CON-O and FMT-O groups (Figure 4I). Nevertheless, no statistically significant difference were found in the levels of SOD and CAT in serum (Figures 4E,H). Thus, the levels of NO and VK2 in serum in CON group were found significantly higher than that in CON-O and FMT-O groups, the level of ET-1 in serum in CON group was found significantly lower than that in CON-O and FMT-O groups (Figures 4J,K,N). Nevertheless, statistically significant difference were found in the level of AngII and VEGF in serum among the three groups (Figures 4L,M). These results suggest that intestinal aging process in mice is related to gut microbiota.

### Significant change of gut microbial diversity was also found in older mice, which was transferable by FMT

**Figure 5:**
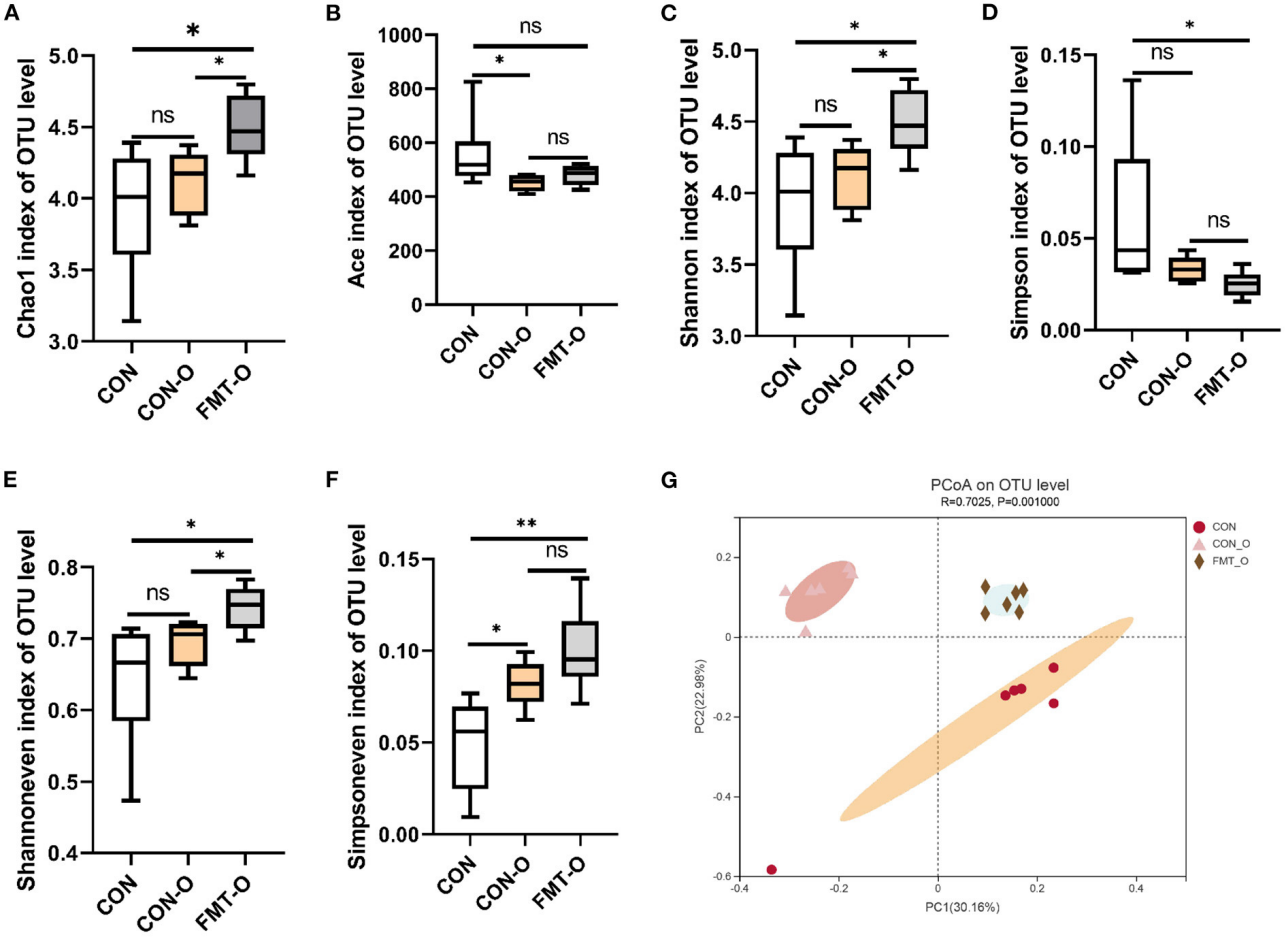
Significant change of gut microbial diversity was found in older mice, which was transferable by fecal microbiota transplantation. The changes of α-diversity **(A–F)**. PCoA plot in β-diversity **(G)**. The closer two sample points are, the more similar the composition of the two sample species. Different colored points and shapes indicate samples from different groups. All data are expressed as mean ± standard error (SEM). One-way ANOVA with Tukey *post hoc* test was conducted. *p < 0.05, **p < 0.01; ns, no significance.

To test the role of gut microbiota in intestinal aging process in mice, we observed the changes in gut microbial diversity. As for alpha diversity, we found the indices of ace and simpson in CON group were evidently higher than that of the CON-O and FMT-O groups (Figures 5B,D). Nevertheless, neither statistically significant difference were found in the index of ace between CON and FMT-O groups, nor in the index of simpson between CON and CON-O groups (Figures 5B,D). The indices of chao1, shannon, shannoneven and simpsoneve in CON group were evidently lower than that of the CON-O and FMT-O groups (Figures 5A,C,E,F). However, there were no statistical difference in the indices of chao1, shannon, shannoneven between CON and CON-O groups, (Figures 5B,D). Indeed, the changes of intestinal aging process in mice are related to gut microbiota according to the changes of gut microbial alpha diversity.

Based on the principal co-ordinates analysis (PCoA) in beta diversity, PC1 accounted for 30.16% of the total variation and PC2 accounted for 22.98% (Figure 5G). The distance in PCoA between the CON and CON-O groups was relatively close, which revealed the microbial community of the CON group differed significantly from that of the CON and CON-O groups (*R*^2^ = 0.7025, *p* = 0.001; Figure 5G). All these findings indicate intestinal aging process in mice is related to gut microbiota.

### Significant change of gut microbial composition was also found in older mice, which was screened by FMT

**Figure 6:**
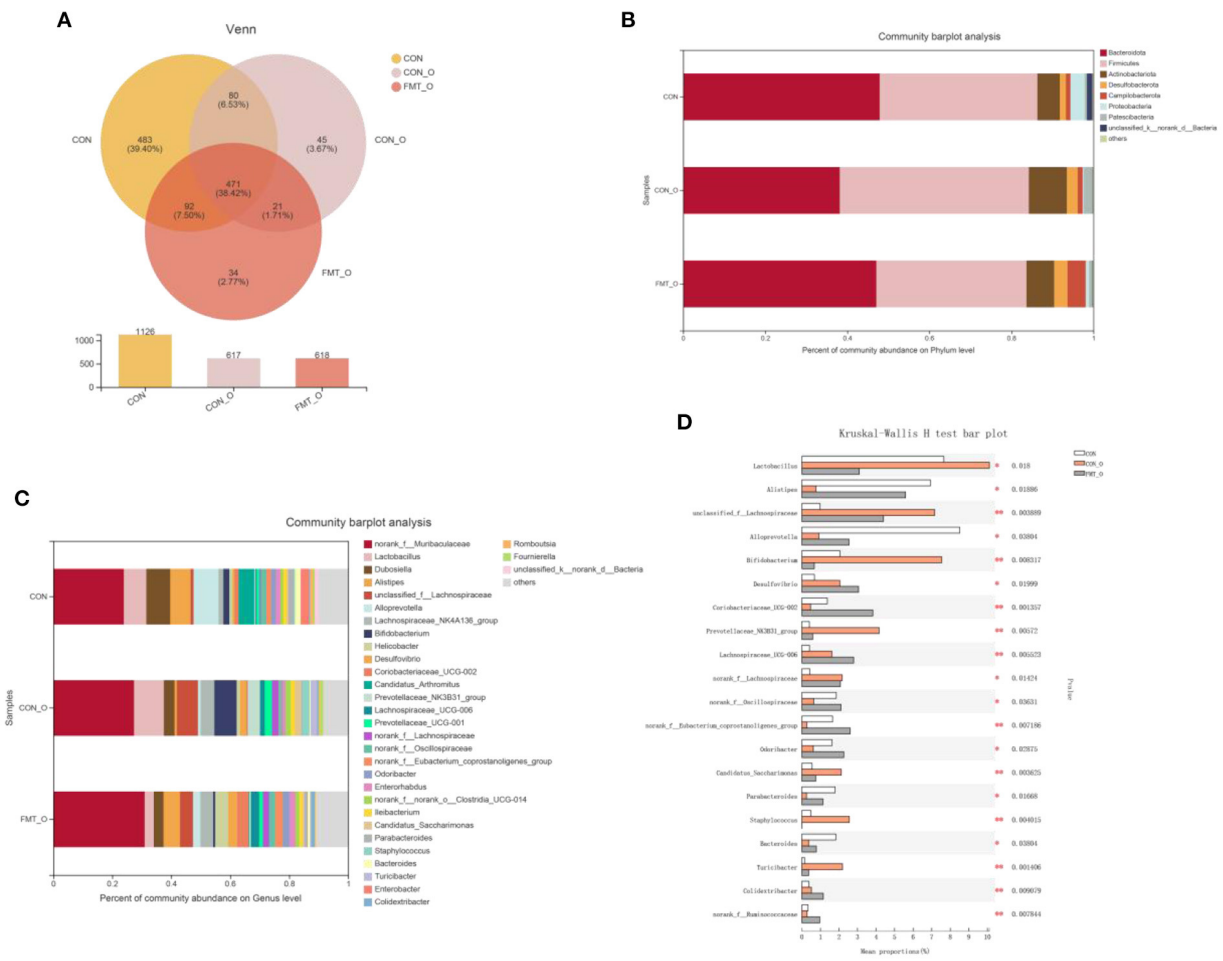
Significant change of gut microbial composition was found in older mice, which was screened by fecal microbiota transplantation (*n* = 6). The down-regulated OTU number is shown in here **(A)**. Phylum-level abundance as a percentage of total community abundance **(B)**. The top 30 in community abundance in terms of genera percent **(C)**. The significantly different genera **(D)**. Kruskal-Wallis test with Dunn *post hoc* test was used for statistical tests. *p < 0.05, **p < 0.01, ***p < 0.001.

To investigate how the microbial characteristics associated with the intestinal healthy aging in mice, we calculated the differential abundance of characteristics among CON, CON-O and FMT-O groups. We found that the 1,126, 617, 618 OTUs in CON, CON-O, and FMT-O group (Figure 6A). The top phyla-level and genera-level were shown as in Figures 6B,C. There were 38.43%, 46.10%, 36.60% Firmicutes in the CON, CON-O, and FMT-O groups, while there were 47.89%, 38.14%, 36.60% Bacteroidetes in the CON, CON-O, and FMT-O groups. In Figure 6C, the top 30 genera were analyzed in the CON, HS, and FMT-HS groups. Such as 23.91%, 27.38%, 30.99% *norank_f__Muribaculaceae* were found in the CON, CON-O, and FMT-O groups; meanwhile, 8.07%, 3.65%, 3.30% *Dubosiella* were found in the CON, HS and FMT-HS groups. Our data indicate that there was difference in the gut microbial community abundance in intestinal aging process. To further investigate the changes of gut microbial composition in intestinal aging process in mice, a total of 52 significantly different genera of the microbial community were found among the three groups (Supplementary Table 2), and the top 20 were shown as Figure 6D. Taken together, all these results reveal the differential abundance of microbial characteristics exist in the intestinal aging process in mice.

### HS significantly induced intestinal aging-related factors imbalance, in which microbiota genera with relative abundance difference were screened by FMT and verified in older mice

**Figure 7:**
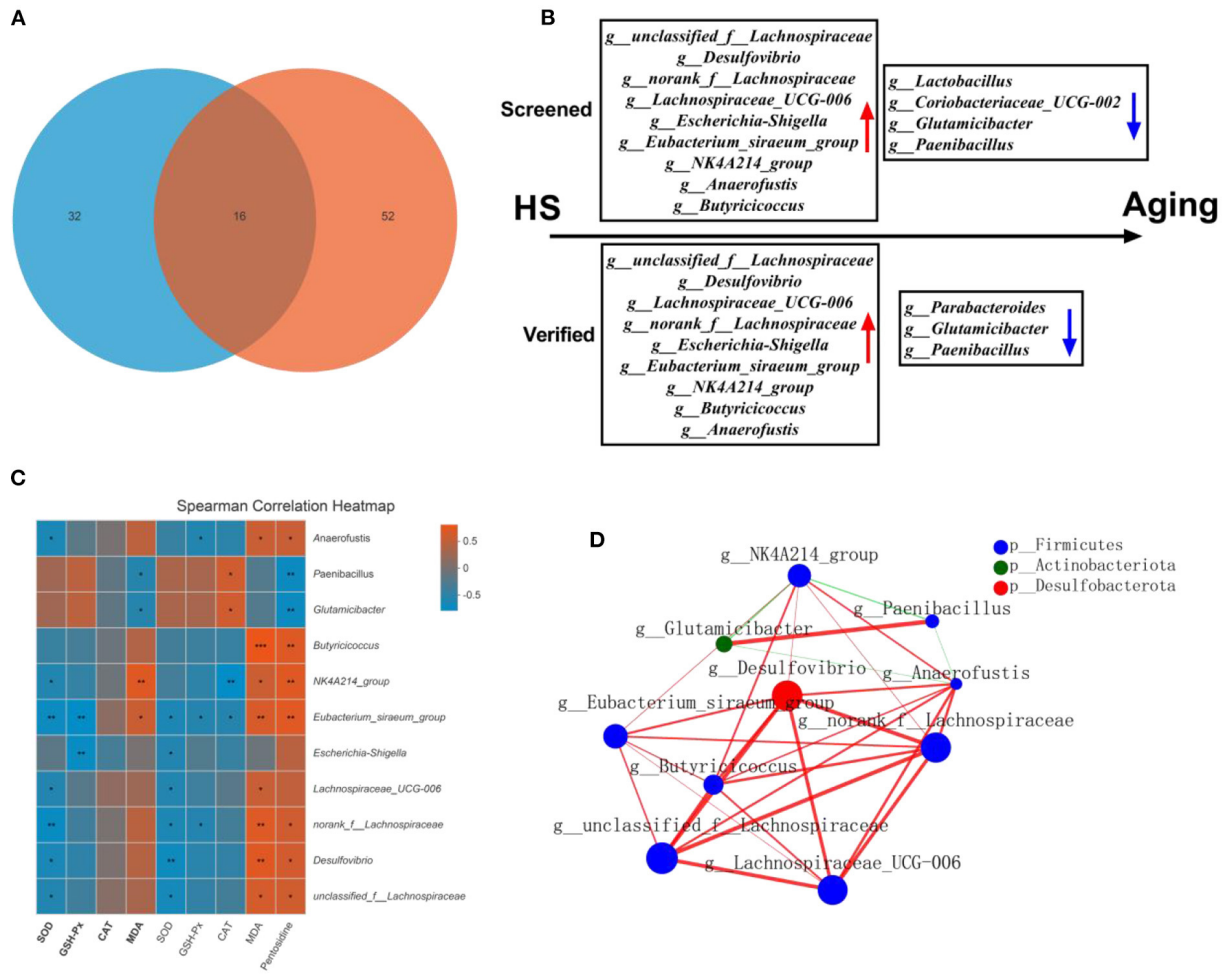
High salt (HS) significantly induced intestinal aging-related factors imbalance, in which key genera were screened by fecal microbiota transplantation and verified in older mice (*n* = 6). The common genra are shown in here **(A)**. The screened and verified gut microbiota in the process of HS induced intestinal aging **(B)**. The correlation between significantly different 11 in community abundance in terms of genera and intestinal aging-related factors **(C)**. The network of the significantly different genera **(D)**. *p < 0.05, **p < 0.01, ***p < 0.001.

As gut microbial compositions were associated with aging status, we sought to investigate the microbial features observed in HS induced intestinal aging mice. To achieve that, we found the 16 common different genera in HS-related 32 significantly different genera (Supplementary Table 1) and age-related 52 significantly different genera (Supplementary Table 2; Figure 7A). In particular, a total of 8 genera with increased relative abundance (such as *unclassified_f__Lachnospiraceae, Desulfovibrio, norank_f__Lachnospiraceae, Lachnospiraceae_UCG-006, Escherichia-Shigella, Eubacterium_siraeum_group, NK4A214_group, Anaerofustis, Butyricicoccus*) and 4 genera with decreased relative abundance (*Lactobacillus, Coriobacteriaceae_UCG-002, Glutamicibacter, Paenibacillus*) were screened in HS induced mice (Figure 7B). Additionally, a total of 9 genera with higher relative abundance (*unclassified_f__Lachnospiraceae, Desulfovibrio, Lachnospiraceae_UCG-006, norank_f__Lachnospiraceae, Escherichia-Shigella, Eubacterium_siraeum_group, NK4A214_group, Butyricicoccus, Anaerofustis*) and 3 genera with lower relative abundance (*Parabacteroides, Glutamicibacter, Paenibacillus*) were found in older mice. Intriguingly, a total of 11 genera were screened in HS induced mice and verified in older mice. The correlation analysis showed the close relationship between the 11 genera and the intestinal aging-related factors (Figure 7C). The network of the significantly different genera were conducted as shown in Figure 7D. These results suggest the 11 genera may be the microbiota genera with relative abundance difference in HS induced aging process.

### Prediction model for intestinal aging based on microbiota genera with relative abundance difference

**Figure 8:**
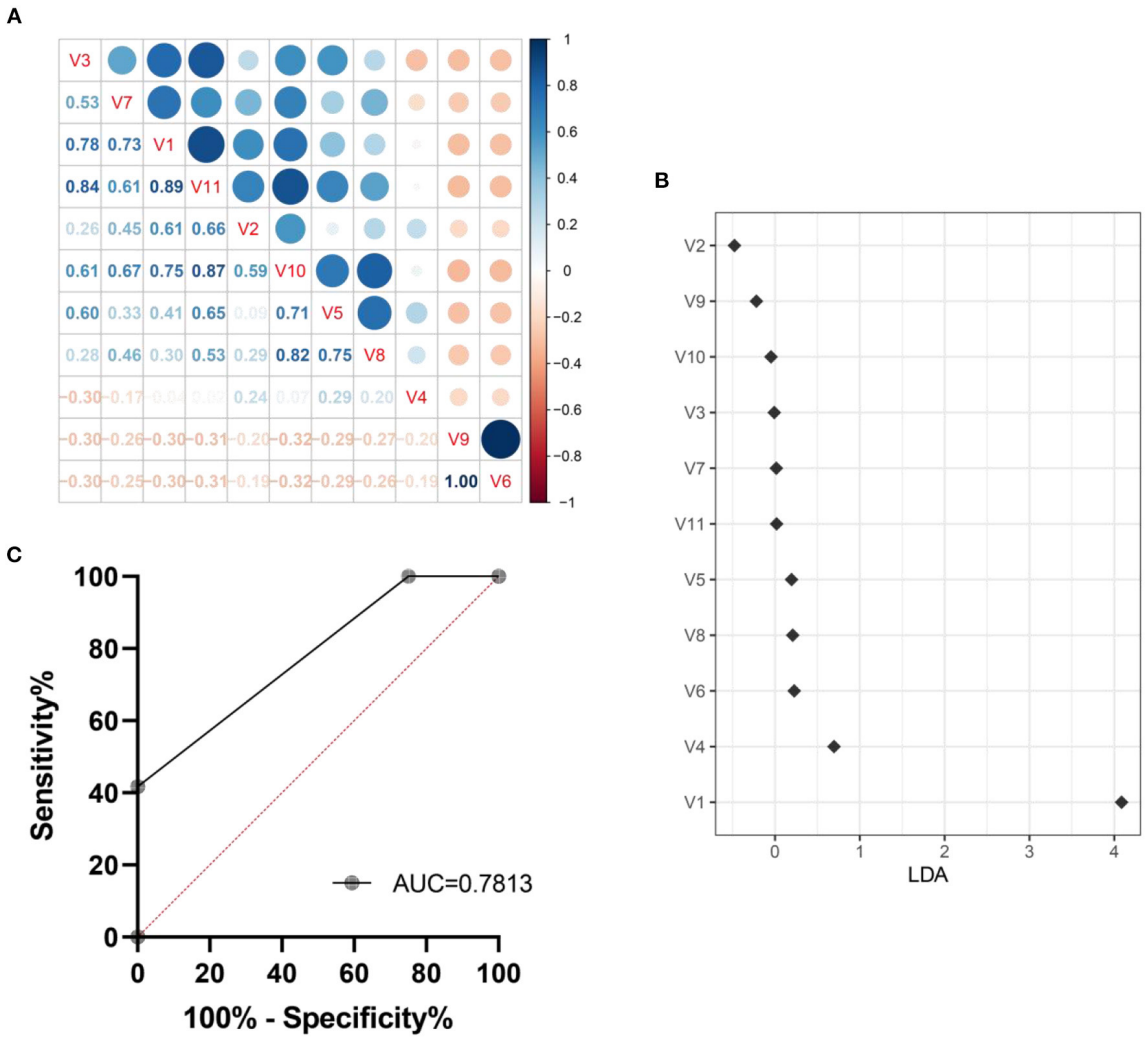
Machine learning model for intestinal aging was constructed and verified based on the significantly different genera in high salt (HS) induced mice. The correlation coefficient among genra are shown in here **(A)**. The LDA linear judgment analysis **(B)**. Evaluation of diagnostic ability based on ROC training set verification **(C)**. V1-V11: *unclassified_f__Lachnospiraceae, Desulfovibrio, Lachnospiraceae_UCG-006, norank_f__Lachnospiraceae, Escherichia-Shigella, Eubacterium_siraeum_group, NK4A214_group, Butyricicoccus, Anaerofustis, Parabacteroides, Glutamicibacter, Paenibacillus*.

We explored a machine learning technique to develop a system for diagnosing intestinal aging based on the 11 genera (Figure 8). The correlation coefficient among the 11 genra are shown in Figure 8A. The data suggest that there is no over fitting of the variables (11 microbiota genera with relative abundance difference) in the model establishment. Moreover, LDA model was established, revealing the contribution of 11 variables (Figure 8B). Thus, we found ROC obtained using only microbiota genera with relative abundance difference in the testing dataset was 0.7813 (95% CI, 0.6536–0.9089, *p* = 0.0008) (Figure 8C). These findings suggest the machine learning model for HS-related intestinal aging based on microbiota genera with relative abundance difference is full of diagnostic ability, which providing better understanding of the complex interaction between HS and intestinal aging.

### Signal transduction pathways enriched by the significantly different genera in HS induced aging process

**Figure 9:**
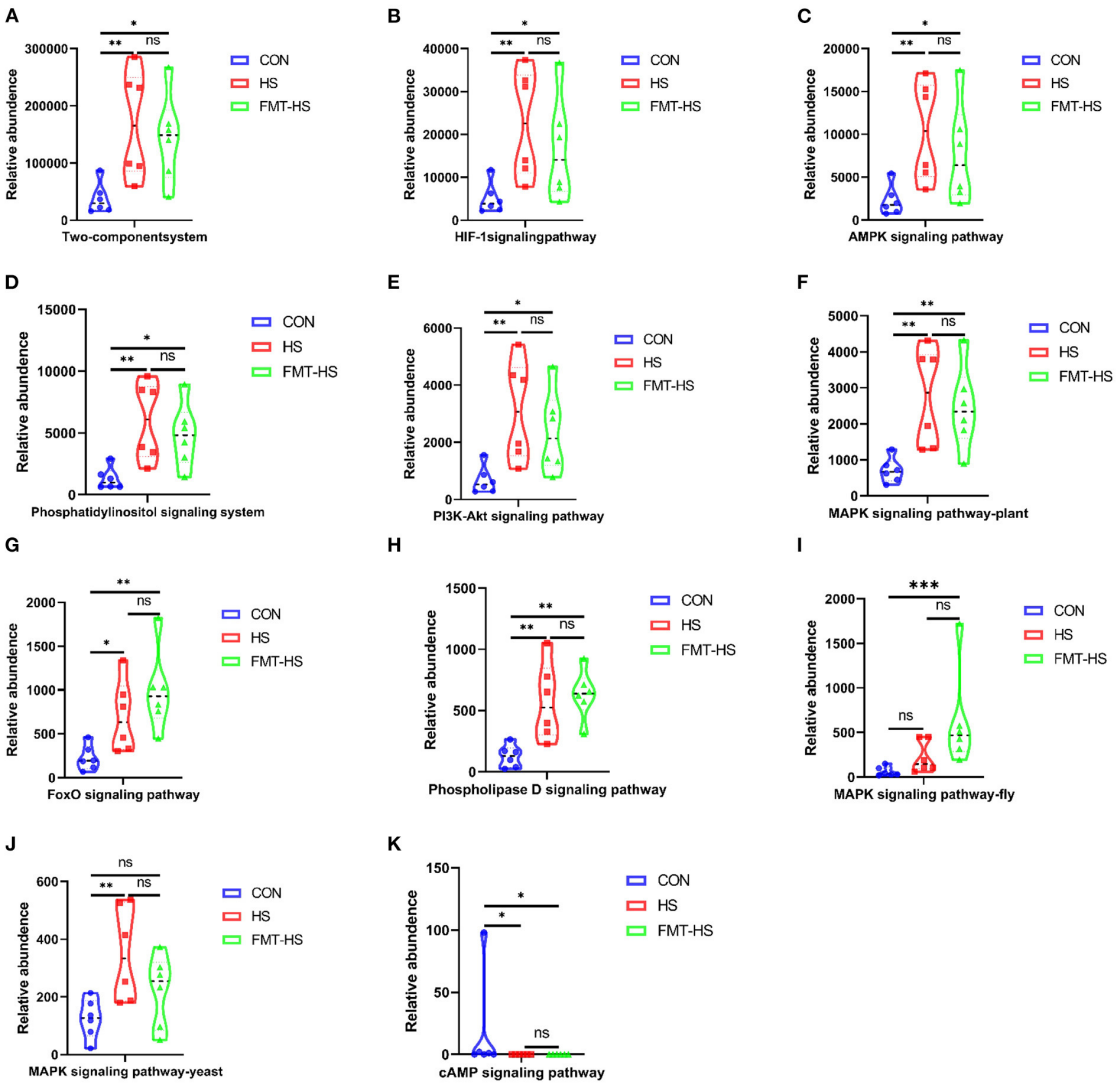
Signal transduction pathways enriched by the significantly different genera in high salt (HS) induced mice which were screened by fecal microbiota transplantation (*n* = 6). The relative abundance of Two-component system, HIF-1 signaling pathway, AMPK signaling pathway, Phosphatidylinositol signaling system, PI3K-Akt signaling pathway, MAPK signaling pathway-plant, FoxO signaling pathway, Phospholipase D signaling pathway, MAPK signaling pathway-fly, MAPK signaling pathway-yeast and cAMP signaling pathway **(A–K)**. *p < 0.05, **p < 0.01, ***p < 0.001; ns, no significance.

**Figure 10:**
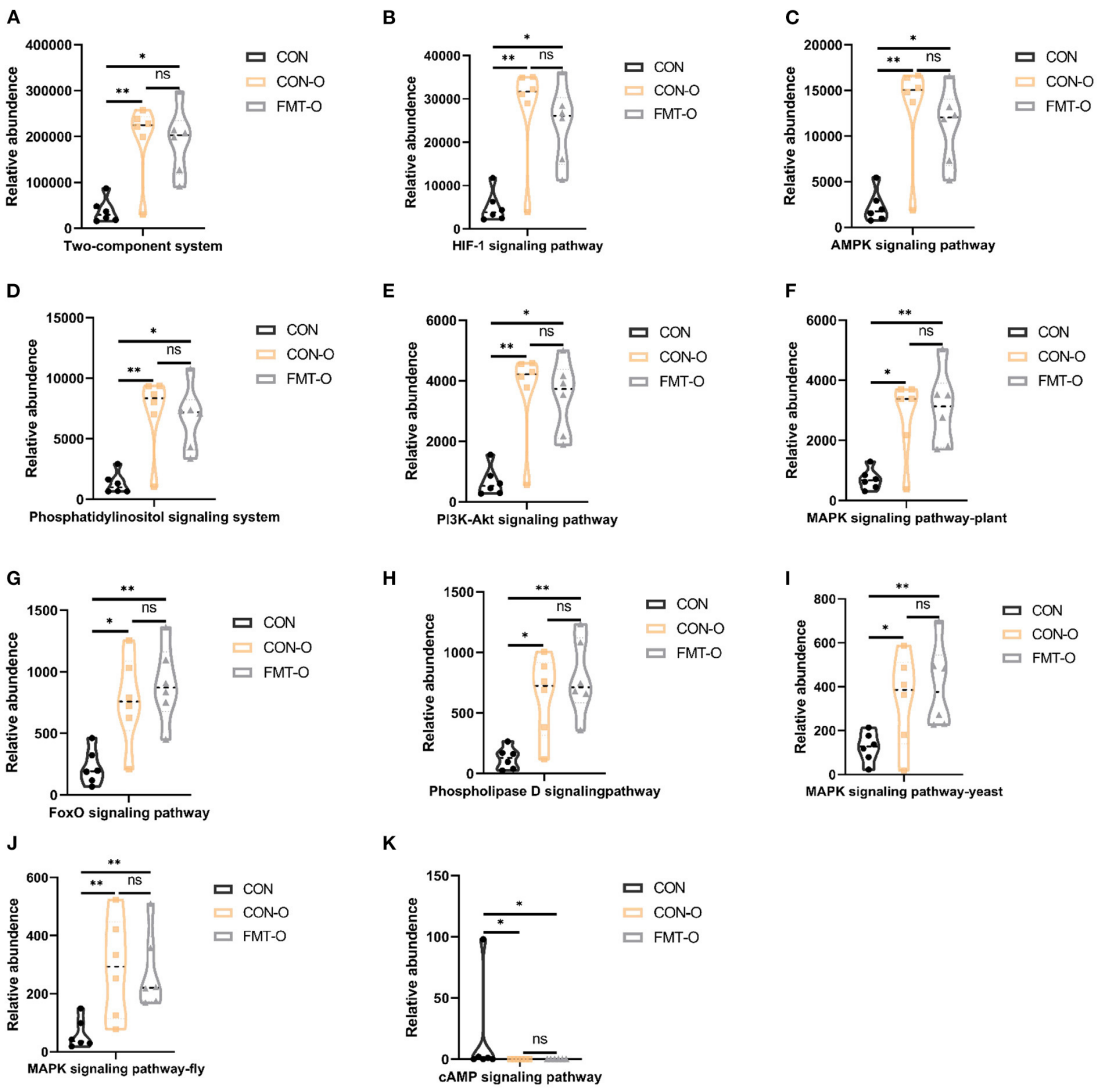
Signal transduction pathways enriched by the significantly different genera in older mice which were screened by fecal microbiota transplantation (*n* = 6). The relative abundance of Two-component system, HIF-1 signaling pathway, AMPK signaling pathway, Phosphatidylinositol signaling system, PI3K-Akt signaling pathway, MAPK signaling pathway-plant, FoxO signaling pathway, Phospholipase D signaling pathway, MAPK signaling pathway-fly, MAPK signaling pathway-yeast and cAMP signaling pathway **(A–K)**. *p < 0.05, **p < 0.01; ns, no significance.

To examine potential gut microbial molecular mechanism, the signal transduction pathways were enriched by the significantly different genera using PICRUSt2. A total of 11 signaling pathways were enriched and identified as shown in Figures 9, 10. Notably, compared with the CON group, increased relative abundance (enriched in the signaling pathways in two-component system, HIF-1 signaling pathway, AMPK signaling pathway, phosphatidylinositol signaling system, PI3K-Akt signaling pathway, MAPK signaling pathway-plant, FoxO signaling pathway, phospholipase D signaling pathway, MAPK signaling pathway-fly, MAPK signaling pathway-yeast and cAMP signaling pathway) was found in HS and FMT-HS groups (Figure 9). However, there was no statistical significance in the relative abundance in MAPK signaling pathway-fly signaling pathway between CON and HS groups (Figure 9I). To better understand these findings, compared with the CON group, higher relative abundance were also significantly enriched in the above pathways in CON-O and FMT-O groups Figure 9. Based on the whole findings, the 11 signaling pathways may be potential gut microbial molecular mechanism in the HS induced aging process.

### Correlation between the significantly different genera and signal transduction pathways

**Figure 11:**
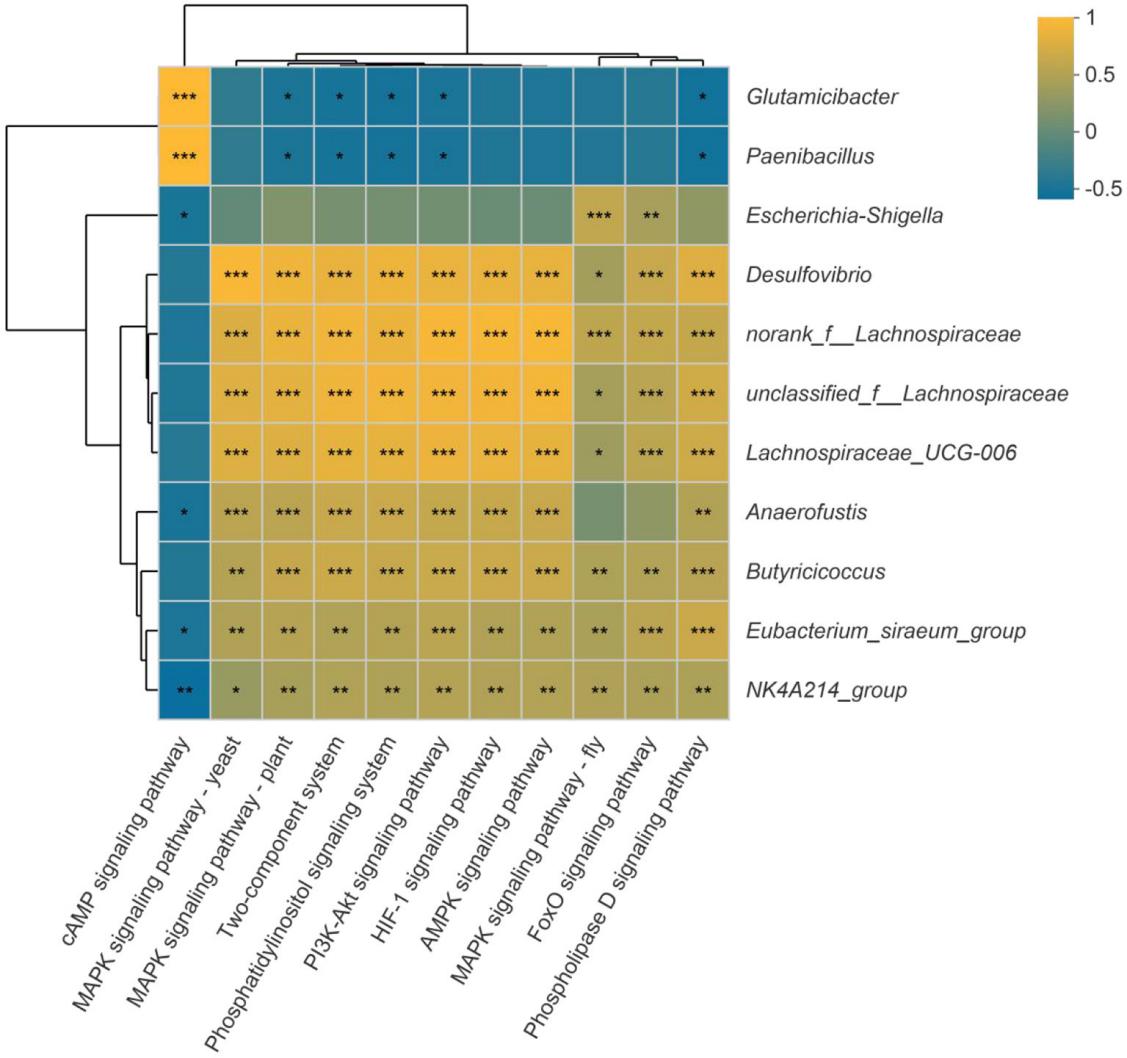
Correlation between the significantly different genera and signal transduction pathways. Dark yellow reveals a positive correlation, while dark blue reveals a negative correlation. *p < 0.05, **p < 0.01, ***p < 0.001.

In addition, the spearman correlation analysis was used to assess the link between the substantially different genera and the signal transduction pathways. As illustrated in Figure 11, *Eubacterium_siraeum_group* and *NK4A214_group* were considerably negatively connected with the cAMP signaling pathway but strongly favorably correlated with all other signaling pathways. With the exception of the cAMP signaling pathway, the other signaling pathways were significantly positively linked with *unclassified_f__Lachnospiraceae, Desulfovibrio, norank_f__Lachnospiraceae* and *Lachnospiraceae_UCG-006*. Furthermore, the two-component system, phosphatidylinositol signaling system, PI3K-Akt signaling pathway, MAPK signaling pathway-yeast, and phospholipase D signaling pathway were strongly connected to 10 of the 11 significantly different genera, which suggest most possible mechanisms of differential bacterial response to host.

## Discussion

Globally, average life expectancy has increased dramatically in recent decades, resulting in a proportionately larger aging population. Although chronological age is currently the most extensively recognized indication of aging, it gives little information on the quality of life during the intestinal aging process. Understanding how to promote healthy intestinal aging will be critical to extending one's life. There is mounting evidence that the gut microbiota is inextricably related to HS and the aging process. Here, mice were fed an 8% HS diet to investigate HS accelerated intestinal aging, and FMT were employed to confirm the association between HS accelerated aging and gut microbiota. Furthermore, young and older mice were utilized to assess gut microbial diversity, and FMT were employed to confirm and discover the crucial function of gut microbiota, as well as to search for evidence of gut microbiota in HS accelerated intestinal aging.

Since oxidative stress-related enzymes are frequently used to evaluate aging, we chose 4 redox enzymes (SOD, GSH-Px, CAT, MDA, pentosidine) as the primary markers for the initial evaluation of aging in this study (13, 14). The increase in cardiovascular disease in aging is partly due to vascular endothelial cell senescence and associated vascular dysfunction, and aging is also often accompanied by vascular endothelial cell aging, so we selected 5 key indicators of vascular endothelial function (NO, ET-1, AngII, VEGF, VK2) to aid in the assessment of aging (12, 15). Advanced glycation end products (AGEs) can continuously accumulate with food intake and self generation, and participate in the progress of aging and related diseases (16, 17). Pentosidine is one of the components of AGEs. VK2 is considered to be related to vascular function in recent years (18, 19), in addition to the role of the latest inhibitor of ferroptosis (20). We evaluated the previous the oxidative stress indicators in the intestinal tissue, serum and the previous vascular endothelial factors in serum. In order to ensure the reliability of the results, we also selected pentosidine and VK2, which reveal that HS diet accelerated the aging of the intestinal tract in mice as much as possible from the biochemical indicators. Consistent with other study (21), our work suggests too much salt may speed up the intestinal aging process.

A prior research (22) similarly showed that the main cause of the chronic hepatic steatosis and inflammation that results in cardiovascular damage under HS loading is SIRT3 suppression induced by histone modification. Additionally, a HS diet promotes lung metastasis, speeds up the formation of breast cancer, and raises the number of Th17 cells in the body. Increased Th17 cells may accelerate the spread of breast cancer by secreting IL-17F, which causes breast cancer cells to activate the MAPK signaling pathway (23). The gut microbiota has been shown in recent years to be intermediate in the physiological responses induced in the host by a high salt diet (24–26). HS diet exacerbates colitis in mice by regulating gut microbial community, especially decreasing *Lactobacillus* levels and butyrate production (27). HS diet regulates a variety of intestinal bacteria, not necessarily all of which can participate in intestinal aging. Therefore, we initially observed the changes of gut microbiota community induced by HS diet, and then conducted preliminary screening and further validation, using the strategy of FMT. FMT can be used to treat recurrent *Clostridium difficile* infection as a therapeutic approach to restore the gut microbiota, since the gut microbiota can partially transfer the intestinal features of the host (28). Furthermore, a great number of clinical trials are investigating the use of FMT in additional disorders associated with the gut microbiota (28). Our previous study used the FMT method to screen and verify the differential microbiota of high salt diet interacting with ATF4 (12). In the current study, our study identified a total of 32 bacterial genera in mouse intestinal contents associated with HS-induced aging that could be significantly associated with aging *via* FMT delivery to control mice. The gut microbiota plays an important role in the physiological succession during the life cycle, and in particular, changes in the gut microbiota are closely related to aging-related diseases, and anti-aging targeting the gut microbiota is an encouraging strategy (29). The current study again reveals that a high salt diet led to intestinal aging in mice associated with gut microbiota, further validating the previous relationship that aging and gut microbiota are closely linked. Previous study suggested that HSD disrupts the balance of the intestinal microbiota primarily by depleting lactic acid-producing bacteria in a dose-dependent manner, and that these are important for salt-sensitive inflammatory diseases (30).

Differences in age are the gold standard for aging. To further characterize the changes in gut microbiota during high salt-induced aging, our study used older mice to reveal evidence of high salt-induced gut microbiota, examining which of the previous 32 bacteria were screened and validated in experiments with older mice. FMT from aged mice to young mice was previously reported to result in disruption of intestinal epithelial barrier integrity, accelerated aging-related systemic inflammation, and reduced levels of key functional visual proteins; in contrast, FMT from young mice reversed these aging-related features in aged mice (31). As in previous studies, our ork also found that FMT from older mice transmitted aging, suggesting that gut microbiota is involved in aging. Finally, this study found that there were 52 significant differences between bacteria and aging in older mice, which illustrate the detailed bacteria in aging process. Combining the two experimental studies before and after, we identified, screened and validated 11 microbiota genera with relative abundance difference (*unclassified_f__Lachnospiraceae, Desulfovibrio, Lachnospiraceae_UCG-006, norank_f__Lachnospiraceae, Escherichia-Shigella, Eubacterium_siraeum_group, NK4A214_group, Butyricicoccus, Anaerofustis, Parabacteroides, Glutamicibacter, Paenibacillus*) associated with high salt-induced intestinal aging, information on which has been elucidated in Figure 7. In this work, a machine learning model for intestinal aging was constructed and verified based on the significantly different genera in HS induced mice, which indicates the 11 microbiota genera with relative abundance difference are of potential diagnostic value in HS-related intestinal aging. Similar to earlier research, our study alos completed the model based on gut microbiota for the first time to evaluate the status of intestinal aging induced by excessive salt (32, 33). Aging is determined by complex interactions among genetic and environmental factors. Increasing evidence suggests that the gut microbiome lies at the core of many age-associated changes, including immune system dysregulation and susceptibility to diseases (11). After studying the mechanisms involved in order to reveal the changes in host function caused by the 11 differential bacteria, we identified 10 signaling pathways (two-component system, HIF-1 signaling pathway, AMPK signaling pathway, phosphatidylinositol signaling system, PI3K-Akt signaling pathway, MAPK signaling pathway-plant, FoxO signaling pathway, phospholipase D signaling pathway, MAPK signaling pathway-fly, MAPK signaling pathway-yeast and cAMP signaling pathway) that were significantly differentially enriched in this process, suggesting that these signaling pathways are associated with HS-induced intestinal aging. Moreover, *Eubacterium_siraeum_group* and *NK4A214_group* were considerably negatively connected with the cAMP signaling pathway but strongly favorably correlated with all other signaling pathways, which reveal *Eubacterium_siraeum_group* and *NK4A214_group* may be the core bacteria in HS related aging. Decreasing *Eubacterium_siraeum_group* has been found in *Lactobacillus plantarum* (LP)-derived postbiotics on ameliorating *Salmonella*-related neurological dysfunctions (34). trans-anethole impaired intestinal barrier and intestinal inflammation was also found associated with *NK4A214_group* (35). Additionally, the two-component system, phosphatidylinositol signaling system, PI3K-Akt signaling pathway, MAPK signaling pathway-yeast, and phospholipase D signaling pathway were considered as the most possible mechanisms of differential bacterial response to host in HS related intestinal aging. Notably, two-component system (36), phosphatidylinositol signaling system (37), PI3K-Akt signaling pathway (38), MAPK signaling pathway-yeast (39), phospholipase D signaling pathway (40) involve the regulation of physiological and pathological activities.

Dietary intervention is regarded as a low-cost, broad-spectrum preventative technique for slowing aging (41). Previous research suggests that increasing circulating TMAO levels throughout the aging process may worsen EC and vascular aging, which is likely due to inhibition of SIRT1 expression and increased oxidative stress, and hence activation of the p53/p21/Rb pathway (42). However, although our study systematically revealed relevant evidence of microorganisms in the process of high salt accelerated intestinal aging through observation, screening and validation in two animal experiments, there are several shortcomings here: (1) lack of further validation in clinical experiments. The relevant evidence of gut microbiota we found has been revealed indirectly, but systematic and direct clinical evidence needs to be further investigated. All these considerations will provide clear research ideas for future studies. (2) Lack of further studies at the level of bacterial strains. Our study revealed the characteristics of gut microbiota changes, but it is not possible to isolate the strains yet. These will also provide directions for future studies. Furthermore, it was reported that oral administration of *Akkermansia* (strains) sufficiently ameliorated the senescence-related phenotype in the intestinal systems in aged mice and extended the health span (43). (3) Lack of further molecular biological level testing of molecular mechanisms. In fact, although further molecular mechanisms have been reported, it would be more convincing to perform quantitative or qualitative experimental assays.

## Conclusion

To summarize, we investigated the influence of age-related changes in gut microbiota on the progression of intestinal aging in HS induced mice in order to identify a microbial profile linked with intestinal aging. Our results suggest a potential relationship between specific gut microbiota and HS in intestinal aging status, which encourages further investigation to validate causality and the potential of future microbiota-targeted therapeutics to support healthy intestinal aging. The study concluded that maintaining a healthy gut microbiota is essential for preventing intestinal aging and that both target gut tissue and a healthy microbiota can aid in preventing or delaying the onset of diseases associated with intestinal aging brought on by HS.

## Data availability statement

The data presented in the study are deposited in the NCBI SRA repository, accession number PRJNA885812. The datasets generated and analyzed during the current study are available in the (Supplementary tables) repository, (https://figshare.com/, doi: 10.6084/m9.figshare.21062974).

## Ethics statement

The animal study was reviewed and approved by the Ethics Committee of Jiangnan University approved the animal experiment which was carried out in the university's animal facility (NO. 20211015c0650220).

## Author contributions

T-hL, C-yZ, and Y-zX participated in study design. T-hL and LZ conducted animal experiment operation. C-yZ, T-hL, and Y-lR helped to draft and revise the manuscript. T-hL, X-yL, T-lW, Y-yD, and Y-yS carried out the statistical analysis of data. All authors contributed to the article and approved the submitted version.

## Funding

The study was supported by the National Natural Sciences Foundation of China (82074307 and 82174148), Wuxi Municipal Health Commission Scientific Research Fund Youth Project (Q202106), and Doctoral talent startup fund of Affiliated Hospital of Jiangnan University.

## Conflict of interest

The authors declare that the research was conducted in the absence of any commercial or financial relationships that could be construed as a potential conflict of interest.

## Publisher's note

All claims expressed in this article are solely those of the authors and do not necessarily represent those of their affiliated organizations, or those of the publisher, the editors and the reviewers. Any product that may be evaluated in this article, or claim that may be made by its manufacturer, is not guaranteed or endorsed by the publisher.

## Abbreviations

HS: High salt
FMT: fecal microbiota transplantation
MDA: malondialdehyde
CAT: catalase
GSH-Px: glutathione peroxidase
SOD: superoxide dismutase
NO: nitric oxide
ET-1: endothelin-1
AngII: angiotensin II
VEGF: vascular endothelial growth factor
VK2: vitamin k2
OTUs: operational taxonomic units.
